# Supplementary material for: Completion of the Continuum of Maternity Care in the Emerging Regions of Ethiopia: Analysis of the 2019 Demographic and Health Survey
Source: Int J Environ Res Public Health. 2023 Jul 7;20(13):6320. doi: 10.3390/ijerph20136320 (PMC10342030; doi:10.3390/ijerph20136320)
Supplement: Supplementary file 1 [file ijerph-20-06320-s001.zip › ijerph-2463130-supplementary.pdf]

Table S1: Factors associated with four or more ANC utilisation in the emerging regions of Ethiopia in the bivariable analysis (N: 1431).

Variables	Categories	ANC4+		
		Yes	No	OR (95%CI)
**Maternal age at the time of delivery (years)**	<20	77	173	1
	20-34	293	703	0.77 (0.53-1.12)
	35-49	47	138	0.57 (0.30-1.06)
**Residence**	Rural	303	872	1
	Urban	114	142	3.21 (1.83-5.61) **
**Region**	Afar	108	279	1
	Somali	30	311	0.27 (0.13-0.56) **
	Benishangul	187	182	2.77 (1.59-4.84) **
	Gambela	92	242	1.02 (0.54-1.91)
Marital status	Not married/not living with partner	35	90	1
	Married/living with partner	382	924	1.16 (0.67-1.99)
**Highest level of maternal education**	No education	187	667	1
	Primary	168	251	3.47 (2.38-5.07) **
	Secondary and higher	62	96	5.68 (2.63-12.28) **
**Sex of the head of the household**	Male	305	686	1
	Female	112	328	0.53 (0.36-0.76) **
**Household wealth index**	Poorest	137	637	1
	Poorer	78	133	3.03 (1.51-6.10) *
	Middle	63	92	3.29 (1.69-6.43) *
	Richer	64	77	3.53 (1.84-6.80) **
	Richest	75	75	4.67 (2.40-9.11) **
**Number of children**	1-2	165	334	1
	3-4	100	273	0.58 (0.37-0.91) *
	> = 5	152	407	0.51 (0.34-0.77) *

P-value: * < 0.05, ** < 0.001

Table S2: Factors associated with institutional delivery in the emerging regions of Ethiopia in the bivariable analysis (N: 1431).

Variables	Categories	Institutional delivery		
		Yes	No	OR (95%CI)
**Maternal age at the time of delivery (years)**	<20	119	131	1
	20-34	416	580	0.80 (0.54-1.18)
	35-49	67	118	0.57 (0.30-1.08)
**Residence**	Rural	414	761	1
	Urban	188	68	5.48 (2.60-11.57) **
**Region**	Afar	105	282	1
	Somali	71	270	0.71 (0.34-1.49)
	Benishangul	231	138	4.12 (1.92-8.82) **
	Gambela	195	139	5.87 (3.28-10.51) **
**Marital status**	Not married/not living with partner	71	54	1
	Married/living with partner	531	775	0.32 (0.17-0.61) **
**Highest level of maternal education**	No education	239	615	1
	Primary	238	181	3.20 (1.85-5.54) **
	Secondary and higher	125	33	11.82 (1.95-71.80) *
**Sex of the head of the household**	Male	413	578	1
	Female	189	251	1.31 (0.86-2.01)
**Household wealth index**	Poorest	175	599	1
	Poorer	112	99	3.85 (2.32-6.39) **
	Middle	89	66	3.08 (1.68-5.65) **
	Richer	99	42	7.88 (3.44-18.04) **
	Richest	127	23	18.06 (8.95-36.44) **
**Number of children**	1-2	165	334	1
	3-4	100	273	0.49 (0.34-0.71) **
	> = 5	152	407	0.52 (0.36-0.74) **

P-value: * < 0.05, ** < 0.001

Table S3: Factors associated with postnatal care utilisation within 24 hours in the emerging regions of Ethiopia in the bivariable analysis (N: 1431)

Variables	Categories	PNC within 24 hours		
		Yes	No	OR (95%CI)
**Maternal age at the time of delivery (years)**	<20	79	171	1
	20-34	252	744	0.70 (0.51-0.96) *
	35-49	48	137	0.55 (0.35-0.84) *
**Residence**	Rural	257	918	1
	Urban	122	134	3.24 (1.87-5.63) **
**Region**	Afar	77	310	1
	Somali	30	311	0.41 (0.22-0.77) *
	Benishangul	151	218	2.64 (1.68-4.14) **
	Gambela	121	213	3.54 (2.08-6.02) **
**Marital status**	Not married/not living with partner	47	78	1
	Married/living with partner	332	974	0.60 (0.33-1.07)
**Highest level of maternal education**	No education	147	707	1
	Primary	146	273	3.17 (1.96-5.13) **
	Secondary and higher	86	72	12.12 (6.73-21.85) **
**Sex of the head of the household**	Male	257	734	1
	Female	122	318	0.82 (0.54-1.24)
**Household wealth index**	Poorest	104	670	1
	Poorer	65	146	2.34 (1.30-4.24) *
	Middle	57	98	2.88 (1.53-5.42) *
	Richer	59	82	6.10 (2.26-16.49) *
	Richest	94	56	10.26 (5.41-19.46) **
**Number of children**	1-2	170	329	1
	3-4	68	305	0.54 (0.32-0.93) *
	> = 5	141	418	0.64 (0.43-0.95) *
**Mode of delivery**	Vaginal	350	1042	1
	Caesarean section	29	10	7.86 (1.90-32.48) **

P-value: * < 0.05, ** < 0.001
